# Aberrant activation of Wnt/β-catenin signaling drives proliferation of bone sarcoma cells

**DOI:** 10.18632/oncotarget.4100

**Published:** 2015-05-11

**Authors:** Changbao Chen, Meng Zhao, Aixian Tian, Xiaolin Zhang, Zhi Yao, Xinlong Ma

**Affiliations:** ^1^ Department of Spinal Surgery, Tianjin Hospital, Tianjin, P. R. China; ^2^ Department of Immunology, Tianjin Key Laboratory of Cellular and Molecular Immunology, Key Laboratory of Educational Ministry of China, School of Basic Medical Sciences, Tianjin Medical University, Tianjin, P. R. China; ^3^ Department of Medical Laboratory, Tianjin Hospital, Tianjin, P. R. China

**Keywords:** bone sarcomas, Wnt/β-catenin signaling, autocrine wnt activation, cell proliferation, therapeutic target

## Abstract

Bone sarcomas such as osteosarcoma and chondrosarcoma are frequently refractory to conventional chemotherapy and radiotherapy that exhibit poor prognosis. The Wnt signaling are evolutionarily conserved and implicated in cell proliferation and sarcomagenesis. However, the potential role of the Wnt signaling in bone sarcomas is still unclear. Here we demonstrate aberrant activation of Wnt/β-catenin signaling in bone sarcoma cells, involving an autocrine Wnt signaling loop with upregulation of specific Wnt ligands and receptors. Activation of Wnt/β-catenin signaling with Wnt3a or GSK-3β inhibitor drives the proliferation of bone sarcoma cells, whereas downregulation of activated Wnt signaling with dnTCF4 or siLEF1 suppresses bone sarcoma proliferation and induces cell cycle arrest. Taken together, our findings establish the evidence that aberrant activation of Wnt/β-catenin pathway involving an autocrine Wnt singaling drives the proliferation of bone sarcoma cells, and identify the autocrine activation of the Wnt/β-catenin signaling as a potential novel therapeutic target for bone sarcomas.

## INTRODUCTION

Osteosarcomas and chondrosarcomas are recognized as the most common primary bone sarcomas [1], which involve mesenchymal tissues and exhibit highly heterogeneous histologic and molecular profiles [2]. Accumulated evidence suggests that bone sarcomas are highly resistant to chemotherapy and radiotherapy, and thus surgical resection is considered as the crucial treatment strategy [3]. Despite an increase in knowledge with regard to identification of critical signaling pathways in bone sarcomas, they are characterized by a relatively high morbidity and mortality [4]. Accordingly, novel therapeutic strategies have emerged into the bone sarcoma field by exploring the biology of bone sarcomas and targeting several crucial pathways governing sarcomagenesis and aggressive clinical features [5].

The WNT signaling pathways are evolutionarily conserved and implicated in multiple crucial physiological and pathophysiological events [6]. To date, three Wnt signaling pathways have been characterized including the canonical Wnt/β-catenin signaling, the noncanonical planar cell polarity pathway (Wnt/PCP) and the Wnt/Ca^2+^ pathway [7, 8]. In the canonical Wnt/β-catenin signaling, a Wnt ligand binds to a cell surface receptor complex consisting of the members of Frizzled family and the transmembrane protein LRP 5 and 6 to prevent phosphorylation and degradation of β-catenin by the GSK3β/APC/Axin destruction complex. Subsequently, accumulated nuclear β-catenin binds to the members of TCF/LEF transcription factor family, and then initiates downstream gene expression programs [7]. Emerging data have illuminated that the certain β-catenin/TCF target genes, such as Axin2, c-Myc, and LEF1, appear to be transcriptionally upregulated in a tissue-independent manner, whereas other target genes may be tissue or context specific [8, 9]. Intriguingly, the Wnt signaling is intricately regulated at multiple levels through receptor modulation [10], and feedback-negative regulators such as Axin2 and DKK1 [9, 11]. DKK1, as a Wnt antagonist and a downstream target of the β-catenin/TCF in a negative feedback loop [12], disrupts Wnt-induced Fz-LRP6 complex formation and inhibits canonical Wnt signaling [6, 13]. Another class of Wnt antagonists, FRPs, binds to and sequesters Wnts, blocking both canonical and noncanonical Wnt signaling [14]. Furthermore, the Wnt signaling is involved in the maintenance of normal tissue and the developmental pathways that regulate the self-renewal and differentiation of mesenchymal stem cells (MSCs) [15, 16]. All these findings have implied that the Wnt signaling is implicated in cell fate determination, differentiation, proliferation and apoptosis during development.

Abnormalities in Wnt signaling are implicated in a variety of human tumors by mechanisms such as inactivating aberrations of APC, CTNNB1, or Axin1 (deletion) [17, 18, 19], and through autocrine Wnt signaling recently recognized [20-22]. Recently, we have presented the evidence that nuclear accumulation of β-catenin play a crucial role in chondrosarcoma development without exploration of potential mechanisms [23]. Accumulated evidence suggest that human mesenchymal stem cells exhibit low levels of endogenous Wnt signaling [24], and can differentiate into osteogenic and chondrogenic lineages in response to specific differentiation media [25]. However, the potential role of Wnt/β-catenin signaling in the pathogenesis of bone sarcomas remained poorly elucidated. Based upon these findings, we deeply seek to explore the underlying role of Wnt signaling in bone sarcoma cells and to investigate whether the canonical Wnt signaling is active and an autocrine Wnt signaling loop could be implicated in human bone sarcomagenesis. Our findings have established the evidence that aberrant activation of Wnt/β-catenin pathway, involving an autocrine Wnt singaling loop, drives the proliferation of bone sarcoma cells, and identify the autocrine activation of Wnt/β-catenin signaling as a potential novel therapeutic target for bone sarcomas.

## RESULTS

### Constitutive activation of canonical Wnt signaling in human bone sarcoma cells

**Figure 1:**
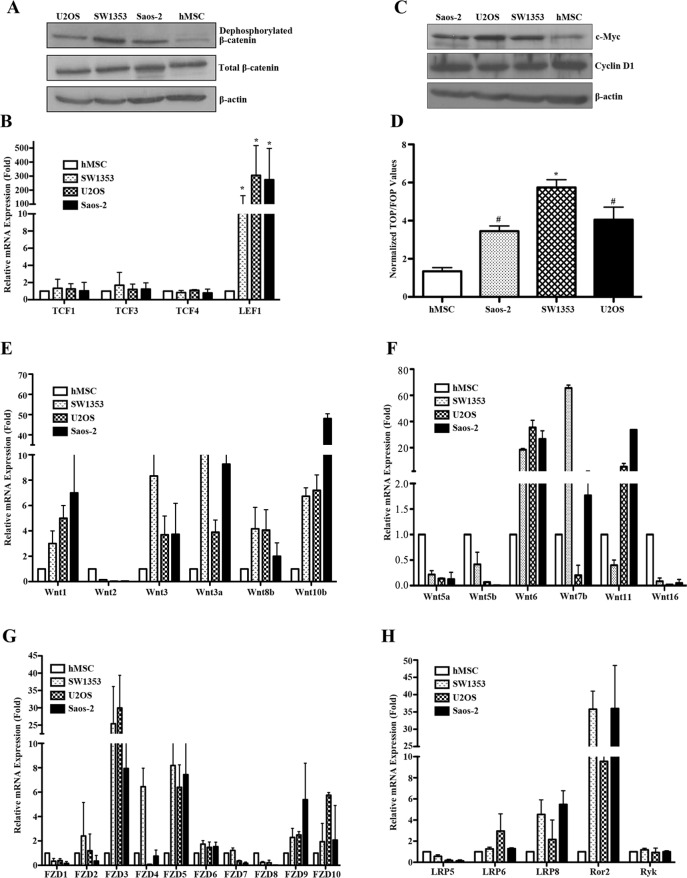
Constitutive activation of canonical Wnt/β-catenin signaling in bone sarcoma cells **A.** Active β-catenin levels were remarkably elevated in bone sarcoma cells as compared to hMSC. Human osteosarcoma cells (U2OS, Saos-2), chondrosarcoma cell (SW1353) and human mesenchymal stem cells (hMSC) were cultured for 24 hr, followed by SDS-PAGE and immunoblot analysis for active β-catenin (anti-ABC, clone 8E7) dephosphorylated on Ser37 or Thr41 (Millipore), total β-catenin (BD Transduction Laboratories) and β-actin (Sigma). β-actin was used as a loading control. **B.** Elevated expression of the LEF1 in bone sarcoma cells compared with hMSC. The indicated TCF transcriptional factor family members were amplified by Real-time PCR in hMSC and bone sarcoma cells. Total RNA from each cell line was reverse transcribed and amplified with primers specific for each indicated gene. Normalized values are represented relative to those in hMSC * *P*<0.01 vs. hMSC. **C.** Increased levels of c-Myc in bone sarcoma cells. The expression of c-Myc, Cyclin D1 and β-actin was assessed by SDS-PAGE and immunoblot analysis. The c-Myc levels were significantly higher in U2OS, Saos-2 and SW1353 cells as compared to hMSC. **D.** Activation of the TCF-reporter transcriptional activity in bone sarcoma cells. U2OS, Saos-2, SW1353 and hMSC cells were cotransfected with either TOP-FLASH or Fop-FLASH plasmid (Upstate Biotechnology), and the pRL-CMV plasmid (Upstate Biotechnology) encoding Renilla luciferase as an internal control for transfection efficiency. Luciferase activity was measured 48 hr after transfection with the Dual-luciferase reporter assay system (Promega). The values represent the mean (±SD) of three independent experiments, and the ratio of the activity obtained with the wild-type TOP-FLASH plasmid to the activity observed with the mutant FOP-FLASH plasmid was shown. Error bars indicate SD of mean values obtained from triplicates and are representative of three independent experiments. *P < 0.01, #*P* < 0.05. **E**-**H.**. Expression profile analysis of the Wnt signaling in hMSC and bone sarcoma cell lines. The relative mRNA levels of canonical Wnt ligands including Wnt1, Wnt2, Wnt3, Wnt3a, Wnt8b and Wnt10b **E.**, noncanonical Wnt ligands including Wnt5a, Wnt5b, Wnt6, Wnt7b, Wnt11and Wnt16 **F.** Frizzled receptors such as FZD1-10 **G.** Co-receptors such as LRP5, LPR6, LPR8, Ror2 and Ryk **H.** were detected by Real-time PCR in hMSC and bone sarcoma cell lines. Total RNA from each cell line was reverse transcribed and amplified with primers specific for each indicated gene. PCR conditions and cycle number were indicated in Table 1. GAPDH is used as an internal control for equal cDNA amount. Normalized values are represented relative to those in hMSC.

The Wnt signaling is mediated through the β-catenin dependent canonical pathway and the β-catenin independent pathway [6-8]. To identify the underlying role of canonical Wnt signaling in human bone sarcomas, we screened hMSCs, SW1353, U2OS and Saos-2 for canonical Wnt activity. As shown in Figure 1A-1D, we have detected upregulated canonical Wnt activity relative to that observed in hMSCs, as evidenced by increased levels of active β-catenin, LEF1, TCF transcriptional activity and downstream target gene c-Myc in all bone sarcoma cell lines. Emerging evidence has demonstrated that there are 19 known genes encoding canonical and noncanonical Wnt ligands [6]. We studied the expression profiles of human Wnt ligands including canonical ligands such as Wnt1, Wnt 2, Wnt3, Wnt3a, Wnt8b and Wnt10b, noncanonical ligands such as Wnt5a, Wnt5b, Wnt6, Wnt7b and Wnt11, and Wnt ligands not clearly established such as Wnt16. As shown in the relative mRNA amount of Figure 1E-1F, the bone sarcoma cells had significantly higher levels of the group of canonical Wnt ligands with exception of Wnt2 compared with hMSC. In contrast, among noncanonical Wnt ligands, the expression of Wnt5a, Wnt5b and Wnt16 was either decreased or lost in bone sarcoma cells; however, Wnt6, Wnt7b, and Wnt11 were remarkably increased in bone sarcoma cells. These findings indicated that bone sarcoma cell lines were equipped with the differential expression of several canonical and noncanonical Wnt signaling ligands, and Wnt ligand expression in bone sarcoma cells is dominated by canonical Wnt ligands, strongly suggesting a ligand-dependent activation of canonical Wnt/β-catenin signaling in human bone sarcomas.

Next, we analyzed the expression of the Wnt receptors and co-receptors in hMSC and bone sarcoma cell lines by Real-time PCR. We found that FZD3, FZD5, FZD9 and FZD10 were prominently expressed in all bone sarcoma cells as compared to hMSC (Figure 1G). LRP6, LRP8 and Ror2 levels were significantly higher in bone sarcoma cells than in hMSC, while LRP5 levels were decreased in bone sarcoma cells (Figure 1H). In addition, Ryk mRNA transcripts remained unchanged in bone sarcoma cells as compared to hMSC. To validate the protein level of Wnt signaling in bone sarcomas, we conducted Western blot and further found that the protein expression levels of the Wnt signaling components were consistent with the relative mRNA amounts as shown in Figure S1. Accordingly, these findings indicated that bone sarcoma cell lines were also equipped with the differential expression patterns of several Wnt receptors, so that each bone sarcoma cell line was likely to respond to both canonical and noncanonical Wnt signals, and play a distinct role in bone sarcomagenesis.

### Autocrine activation of Wnt signaling and functional effects in bone sarcoma cells

**Figure 2:**
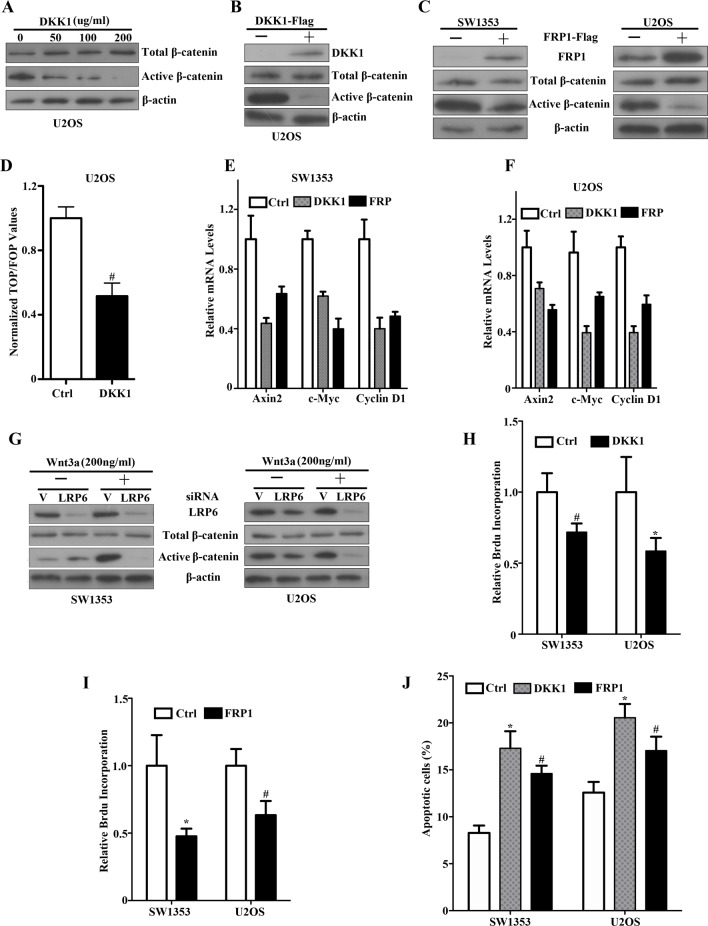
An autocrine Wnt signaling loop by DKK1, FRP1 inhibition and siLRP6 in bone sarcoma cells **A.** Soluble DKK1 inhibits upregulated active β-catenin levels in U2OS cells. Cultures were exposed to increasing concentrations of purified DKK1 (−, 50, 100, 200ng/ml) for 12 hr, solubilized, and analyzed for total β-catenin (top panel), active dephosphorylated β-catenin (middle panel), and β-actin (lower panel). **B.** U2OS cells were transfected with either empty vector or DKK1-Flag. Expression of DKK1, total β-catenin, and active dephosphorylated β-catenin was assessed by immunoblot analysis of lysates with an anti-Flag antibody, anti-total β-catenin and dephosphorylated β-catenin. β-actin was used as a loading control. **C.** SW1353 and U2OS cells were transfected with either empty vector or FRP1-Flag. Expression of tagged FRP1 was assessed by immunoblot analysis of lysates with an anti-Flag antibody (top panel). Total β-catenin (middle panel), active dephosphorylated β-catenin (middle panel) and β-actin (lower panel) were analyzed by western blot. **D.** DKK1 inhibition of TCF-response elements in U2OS cells. Cells were transfected with either empty vector or FRP1-Flag for 48 hr. Cells were then cotransfected with either TOP- or FOP-plasmids, and the pCMV-RL plasmid encoding Renilla luciferase. The values represent the mean (±SD) of two independent experiments, and the ratio of the activity obtained with the wild-type TOP-plasmid to the activity observed with the mutant FOP-plasmid is shown ^#^ *P*<0.05. **E**, **F.** Real-time PCR quantification of DKK1 and FRP1 effects on Axin2, c-Myc, Cyclin-D1 mRNA expression. SW1353 **E.** and U2OS **F.** cells were transfected with vector (Ctrl), DKK1-Flag or FRP1-Flag. qRT–PCR was performed as described in Materials and Methods. Relative mRNA expression levels were quantified. **G.** Effects of LRP6 siRNAs on Wnt3a-activated canonical Wnt/β-catenin signaling Depletion of LRP6 by specific siRNAs in SW1353 (left panel) and U2OS (right panel). siRNAs targeting human LRP6 were purchased by SignalSilence LRP6 siRNA (#8650). Bone sarcoma cells were transiently transfected with 2 ug of siRNA construct. At 48 hr, LRP6 levels were detected utilizing an LRP6 antibody. Effects of LRP6 siRNAs on Wnt-3a-stimulated bone sarcoma cells in SW1353 (Left, middle and lower panel) and U2OS (Right, middle and lower panel). Subconfluent bone sarcoma cells were transiently transfected with 2 ug of siRNA construct. After 48 hr, cells were treated for two hours with either control or Wnt3a conditioned media and analyzed for total β-catenin, active dephosphorylated β-catenin levels. The same lysates were analyzed with a β-actin antibody as a loading control. **H**, **I.** Inhibition effects of DKK1 and FRP1 on cell proliferation by Brdu incorporation assay. SW1353 and U2OS cells were transfected with either empty vector or DKK1-Flag (**H**) or FRP1-Flag (**I**). Cell proliferation was determined by Brdu incorporation assay. ^#^ *P* <0.05, * *P* <0.01. J. Enhancement effects of DKK1 and FRP1 on apoptosis in response to a chemotherapeutic drug. Subconfluent cultures of SW1353 and U2OS cell lines were transfected with either empty vector or DKK1-Flag or FRP1-Flag for 48 hr, and then treated with the 1μg/ml of cisplatin for 24 hr. The apoptotic cells were determined by FACS analysis ^#^ *P* <0.05, * *P* < 0.01.

Previously, we have presented the evidence that DKK1 levels were remarkably elevated in chondrosarcoma specimens and DKK1 suppressed canonical Wnt/β-catenin signaling in human chondrosarcoma SW1353 cells [23]. Thus, to directly address whether constitutive Wnt pathway activation in these sarcoma lines involved an autocrine Wnt signaling loop, we took advantage of the DKK1 and FRP1 antagonists. As shown in Figure 2A, exposure of U2OS cells to increasing concentrations (0-200μg/ml) of recombinant DKK1 protein led to a dose-dependent, dramatic reduction in the levels of active β-catenin while the total β-catenin remained unchanged. Overexpression of DKK1 or FRP1 in U2OS and SW1353 cells can also result in a marked reduction in active β-catenin levels (Figure 2B, 2C). Furthermore, DKK1 caused a striking reduction in the level of TCF-responsive transcription in U2OS cells (Figure 2D). We did observe significant reduction in the levels of Axin2, c-myc and Cyclin D1 (Figure 2E, 2F). These findings showed canonical Wnt signaling inhibition in response to DKK1 or FRP1, supporting an autocrine loop of Wnt signaling activation in these sarcoma lines, and further established that TCF-dependent transcription was constitutively activated in such bone sarcoma cells by an autocrine Wnt mechanism.

In an effort to independently confirm the existence of an autocrine Wnt loop in bone sarcoma cells, we generated siRNAs directed against LRP6 that was markedly higher in bone sarcoma cells as shown in Figure 1H, the Wnt co-receptors specific for the canonical pathway. When the specific siRNAs were expressed in SW1353 cells treated with Wnt3a stimulation, we observed that LRP6 siRNA caused a dramatic reduction in Wnt3a induced active β-catenin levels (Figure 2G, left panel). These results implied that canonical signaling in response to Wnt3a required endogenous LRP6. We next tested the effects of the same siRNAs on U2OS cells and observed that LRP6 siRNA caused a marked inhibition in active β-catenin levels as well (Figure 2G, right panel). Therefore, these results provide strong evidence, independent of Wnt antagonists, that constitutive activation of Wnt signaling involved an autocrine Wnt loop, and implicated LRP6 as the specific Wnt canonical co-receptor involved in bone sarcomas.

Having identified bone sarcoma cells with autocrine Wnt signaling activation, we analyzed the functional effects of autocrine Wnt signaling inhibition on the phenotypes of sarcoma cells. As shown in Figure 2H and 2I, SW1353 and U2OS cells overexpressing DKK1 or FRP1 exhibited a marked reduction of cell proliferation when compared to vector-transduced parental cells. To investigate the effects of Wnt inhibition by FRP1 or DKK1 on the response of U2OS and SW1353 cells to apoptotic stimuli, we exposed the cells to 1μg/ml of cisplatin for 24 h, and analyzed the apoptotic response. As expected, there was a statistically significant increase in the level of apoptosis in the presence of DKK1 or FRP1 overexpression (Figure 2J). All of these findings provide evidence for an autocrine Wnt transforming mechanism via driving cell proliferation and attenuating apoptosis of bone sarcoma cells.

### Functional effects of canonical Wnt/β-catenin signaling activation in bone sarcomas

**Figure 3:**
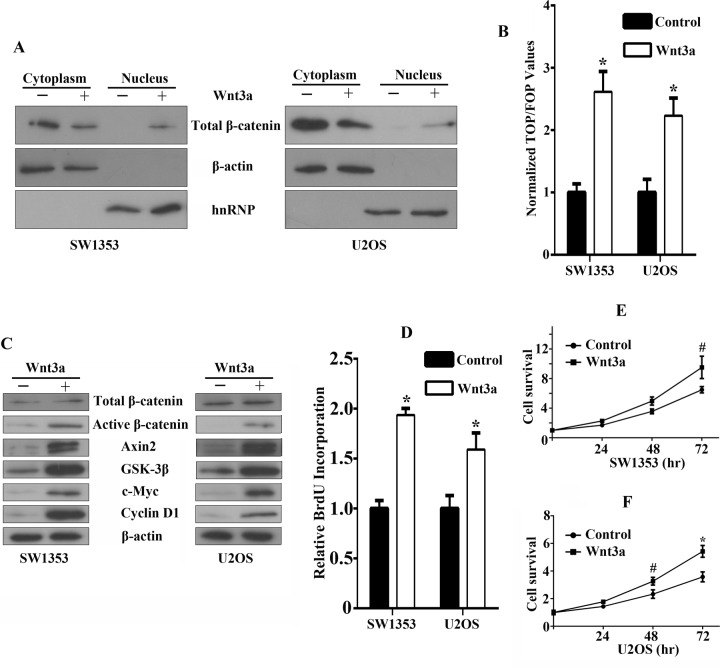
Functional effects of canonical Wnt/β-catenin signaling activation in bone sarcoma cells **A.** β-catenin accumulation and nuclear translocation in bone sarcoma cells in response to Wnt3a. SW1353 and U2OS cells were incubated for 12 hr in the absence or presence of 200ng/ml Wnt3a. To assess β-catenin nuclear accumulation, cells were lysed and were immunoblotted by using a monoclonal anti-β-catenin antibody (Top). The middle and bottom part of the blot was stained with anti-β-actin or hnRNP to verify equal loading. **B.** Activation of the TCF-reporter transcriptional activity in bone sarcoma cells in response to Wnt3a. SW1353 and U2OS cells were incubated for 12 hr in the absence or presence of 200ng/ml Wnt3a, and then the TCF-reporter transcriptional activity was determined by luciferase activity as indicated in Materials and Methods. **C.** Western blot analysis of Wnt3a effects on total β-catenin, active dephosphorylated β-catenin, Axin2, GSK-3β, c-Myc, Cyclin-D1, and β-actin protein expression. SW1353 and U2OS cells were incubated for 12 hr in the absence or presence of 200ng/ml Wnt3a. The whole-cell lysates were immunoblotted with the indicated antibodies. **D.** Effect of Wnt3a on the cell proliferation by Brdu incorporation assay. SW1353 and U2OS cells were incubated for 48 hr in the absence or presence of 200ng/ml Wnt3a. Cell proliferation was determined by Brdu incorporation assay as indicated in Material and Methods. **E**, **F.** Effect of Wnt3a on the cell survival by MTS assay. SW1353 **E.** and U2OS **F.** cells were incubated for 24, 48, 72 hr in the absence or presence of 200ng/ml Wnt3a. Cell survival was determined by MTS assay as indicated in Materials and Methods ^#^ *P* < 0.05, * *P* < 0.01.”.

In order to assess whether human recombinant Wnt3a treatment affects β-catenin, in particular its intracellular redistribution, we performed western blot analysis and found that Wnt3a stimulation resulted in nuclear translocation of a fraction of β-catenin with fractioned extracts, while the amount of control nuclear protein hnRNP remained unchanged (Figure 3A). After Wnt3a stimulation, normalized TOP/FOP values were remarkably increased compared with untreated cells, indicating significant induction of β-catenin-driven TCF/LEF luciferase activity (Figure 3B). As expected, immunoblot analysis for downstream Wnt targets in Wnt3a-treated cell lines showed significant elevation in active β-catenin, Axin2, GSK-3β, c-Myc, Cyclin D1 expression, whereas the expression of total β-catenin remained unchanged (Figure 3C). We also observed a marked increase in proliferation rates of SW1353 and U2OS cells after stimulation of Wnt3a (Figure 3D). In addition, Wnt3a stimulation can readily promote bone sarcoma cell survival (Figure 3E, 3F). Therefore, these data indicate that the canonical Wnt/β-catenin signaling is functionally active in bone sarcoma cells, driving cell proliferation and promoting survival.

### GSK-3β inhibition enhances the survival of bone sarcoma cells by attenuating apoptosis

**Figure 4:**
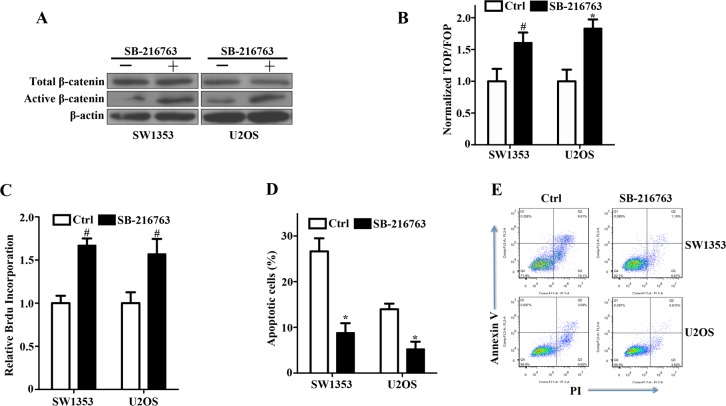
Activation of canonical Wnt/β-catenin signaling and enhancement of bone sarcoma cell survival by a GSK-3β inhibitor **A.** GSK-3β inhibitor SB-216763 increased active β-catenin levels by inhibiting GSK-3β activity in SW1353 and U2OS cells. Bone Sarcoma cells were incubated with 10ng/ml SB-216763 or the DMSO vehicle for 24 hr. The whole-cell lysates were immunoblotted with the indicated antibodies. **B.** Activation of the TCF-response elements by SB-216763. SW1353 and U2OS cells were treated with the GSK-3β inhibitor SB-216763 (10ng/ml) or the DMSO vehicle for 24 hr, and then the TCF-reporter transcriptional activity was determined by luciferase activity as indicated in Materials and Methods. **C.** The prosurvival activity of SB-216763 in SW1353 and U2OS cells. Bone sarcoma cells were incubated with 10ng/ml SB-216763 or the DMSO vehicle for 24 hr before determinations of cell proliferation by relative Brdu incorporation assay. The mean incremental survivals measured in triplicate and the SD were shown. **D.** SB-216763 protected bone sarcoma cells from apoptosis induced by cisplatin. SW1353 and U2OS cells were incubated with 10ng/ml SB-216763 or the DMSO vehicle for 24 h, and then treated with 1μg/ml cisplatin for 24 h. The apoptotic cells were measured by flow cytometry by using the FITC-conjugated Annexin V and PI. Three independent experiments were analyzed. **E.** Two representative bone sarcoma cells of the anti-apoptotic effect of SB-216763 were shown by the FITC-conjugated Annexin V and PI ^#^ *P* < 0.05, * *P* < 0.01.

The phosphorylation of β-catenin by GSK-3β marks it for ubiquitination and degradation [9]. Thus, inhibition of this activity allows β-catenin to accumulate in the nucleus and activate TCF/LEF-dependent transcription. In bone sarcoma SW1353 and U2OS cells, the GSK-3β inhibitor SB-216763 substantially upregulated active β-catenin while total β-catenin had no detected change (Figure 4A), and enhanced reporter gene activity (Figure 4B). Moreover, incubation of SW1353 and U2OS cells with the GSK-3β inhibitor improved the survival in culture (Figure 4C), and attenuated apoptotic cell death (Figure 4D, 4E). These findings have demonstrated that activation of Wnt/β-catenin signaling with a GSK-3β inhibitor enhanced the survival of bone sarcoma cells by attenuating apoptosis.

### Disruption of activated Wnt signaling suppresses the proliferation of bone sarcoma cells

**Figure 5:**
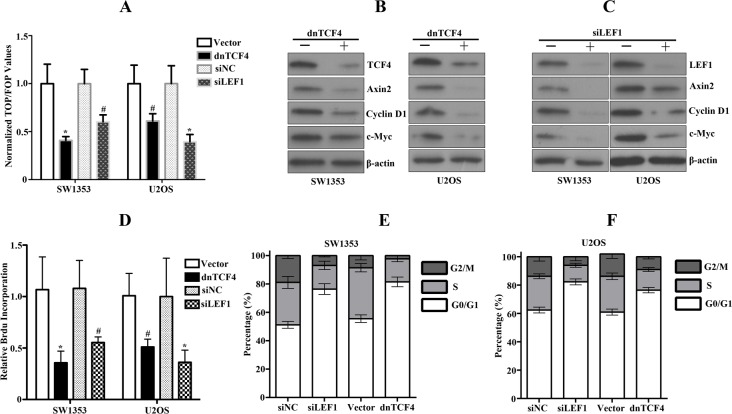
Disruption of activated Wnt signaling by dnTCF or siLEF1 in bone sarcoma cells induces inhibition of cell proliferation **A.** Downregulation of Wnt signaling in bone sarcoma cells by dnTCF4 or siLEF1 was assessed by TCF-response elements activity. Error bars indicate SD of mean values from triplicates and are representative of three independent experiments ^#^ *P* < 0.05, * *P* < 0.01. **B**, **C**. Cell lysates from bone sarcoma cells transduced with dnTCF4 or transfected with siLEF1 were subjected to immunoblot analysis for TCF4, LEF1, Axin, Cylin D1, c-MYC, and β-actin. **D**. Human bone sarcoma cells were transduced with either vector control or dnTCF4, or transfected with siRNA control or siRNA targeting LEF1. *In vitro* cell proliferation was assessed by Brdu incorporation assays. Results are representative of at least two independent experiments. ^#^ *P* < 0.05, * *P* < 0.01. **E, F.** Cell cycle profile analysis of bone sarcoma cells stably expressing dnTCF4 or transfected with siLEF1 performed by FACS.

To further address the biological effects of upregulated canonical Wnt signaling in Wnt autocrine SW1353 and U2OS cells, we transduced sarcoma cells with dnTCF4 or siLEF targeting TCF/LEF signaling. Using this strategy, we observed approximate 50% inhibition of TCF reporter activity (Figure 5A) associated with markedly decreased Wnt downstream target genes such as c-Myc, Axin2 and Cyclin D1 (Figure 5B, 5C). Furthermore, we also detected dramatic reduction of bone sarcoma cell proliferation by dnTCF4 expression or siLEF1 (Figure 5D). Analyses of cell cycle profiles of bone sarcoma cells expressing dnTCF4 or siLEF indicated that growth inhibition was achieved either via G1 or G2 arrest (Figure 5E, 5F). These findings establish that the activation of Wnt/β-catenin pathway significantly contributed to the proliferation of bone sarcoma cells.

## DISCUSSION

Our present findings have established that constitutive activation of the canonical Wnt/β-catenin signaling in human bone sarcoma cells, by a novel mechanism involving an autocrine Wnt signaling loop, drives the proliferation of bone sarcoma cells. Stimulation of Wnt/β-catenin signaling with Wnt3a or GSK-3β inhibitor enhanced the proliferation and survival of bone sarcoma cells, whereas antagonism of this pathway with dnTCF4 or siLEF1 had the exact opposite effect. These data suggest that aberrant activation of Wnt/β-catenin signaling involving an autocrine Wnt signaling loop is frequent event in bone sarcomas and drives cell proliferation. Thus, disruption of autocrine activation of Wnt/β-catenin signaling could be a novel attractive therapeutic target for bone sarcomas.

Previous studies have documented that hMSCs exhibit a low level of endogenous Wnt signaling and can differentiate into osteogenic and chondrogenic lineages [24, 25]. However, exogenous Wnt stimulation can specifically increase hMSC proliferation and inhibit cell differentiation [24], which is commonly characterized by the properties of tumor cells [27]. These data prompted us to explore whether activation of Wnt signaling might contribute to bone sarcomagenesis. As shown in Figure 1, we have detected increased levels of the active dephosphorylated form of β-catenin and TCF transcriptional activity relative to that observed in hMSCs. We also found that the mRNA and protein expression of Wnt ligands is prominently dominated by canonical Wnt ligands in bone sarcoma cells (Figure 1, S1). Accumulated studies have shown that only these dephosphorylated β-catenin species are signaling competent and can transduce Wnt signals to the nucleus [7, 8]. Given the ability of active β-catenin as oncogenic function, upregulated canonical Wnt activity could play a crucial role in the pathogenesis of bone sarcomas. Notably, we found the activation of Wnt/β-catenin signaling in both osteosarcoma and chondrosarcoma cells. The previous study has documented that canonical Wnt signaling influences the maintenance of mesenchymal stem cells, and sarcomas commonly select for upregulation of Wnt signaling [22]. The canonical Wnt pathway is upregulated in a large fraction of human sarcoma cell lines including U2OS and Saos2, and primary sarcoma tissues of diverse subtypes such as 37.5% (3/8) of chondrosarcoma tissues and 50% (3/6) of osteosarcoma tissues [22]. These results were consistent with our findings. Moreover, our previous report had presented that β-catenin levels were remarkably elevated in 53.9% (34/63) of primary chondrosarcoma tissues, and in 17.6% (3/17) of the benign cartilage tumors [23], suggesting that the canonical Wnt activity was activated in primary chondrosarcoma tissues. Thus, the high frequency at which the Wnt pathway is activated in human bone sarcomas identifies Wnt signaling as a potential target for therapies. In contrast, the authors have shown that the Wnt signaling is lost during the peripheral chondrosarcoma progression of grade I towards grade III and is decreased in high-grade peripheral chondrosarcomas [28, 29]. Limited numbers of cases and differences in the methods, assay sensitivity and chondrosarcoma tissues enrolled may explain the difference in the canonical Wnt signaling activity observed in these studies. Regarding as the canonical Wnt signaling activity in osteosarcoma, some osteosarcoma cell lines were found to have relatively increased β-catenin transcriptional activity relative to human MSCs, and increased expression of β-catenin and LEF1 mRNA in human osteosarcoma compared to human fetal osteoblasts, paired with increased active β-catenin protein by western blot [22, 30]. Consistently, the expression of Wnt/β-catenin inhibitors is often suppressed in osteosarcoma including sFrzB and WIF1 [31, 32]. These findings, in combination with our results, imply that abnormal activation of the Wnt signaling plays a role in osteosarcoma formation. However, contradictory to these findings, the Wnt/β-catenin pathway was inactivated in osteosarcoma and that the loss of Wnt-β-catenin activity induces osteosarcoma development [33]. Similarly, Piskun CM et al have demonstrated that β-catenin transcriptional activity was found to be three-fold higher in the normal primary canine osteoblasts relative to these canine osteosarcoma cell lines [34]. Bongiovanni L et al also found increased β-catenin intensity and nuclear localization in canine osteoblasts compared to canine osteosarcoma clinical samples [35]. The inconsistencies of relative β-catenin transcriptional activity in MSCs, osteoblasts and osteosarcoma cells are both a function and a clear representation of the lack of precise knowledge concerning the cell-of-origin for osteosarcoma. However, our results together with the previous study [22], support the notion that Wnt/β-catenin signaling is aberrantly activated in osteosarcoma. Therefore, further studies will be performed to provide direct genetic evidence of this pathway in osteosarcoma pathogenesis.

Several studies have illuminated the antagonism and cross-talk between the canonical and noncanonical Wnt pathways [36, 37]. However, a comprehensive analysis of Wnt signaling components in bone sarcoma cells is lacking. In this study, we found that five of six canonical Wnt ligands (Wnt1, 3, 3a, 8b and 10b) in bone sarcoma cells were prominently increased, while Wnt2 expression was remarkably decreased. However, Park JK et al have reported that Wnt2 is overexpressed in colorectal cancer that plays an oncogenic role in cancer [38]. Thus, it will be important to further investigate the role of Wnt2 in bone sarcomagenesis. In contrast, the noncanonical Wnt6, Wnt7b, and Wnt11 were significantly increased in bone sarcoma cells (Figure 1F, S1). Regarding as the relationship among Wnt6, Wnt7b and Wnt11, the noncanonical Wnt signaling, and tumorigenesis, Galbraith RL et al have reported that the polymorphisms in Wnt6 was associated with increased risk of colorectal adenoma [39], suggesting that Wnt6 may involve in tumorigenesis by initiating the noncanonical Wnt signaling. Wnt7b promotes androgen-independent advanced prostate cancer cell proliferation through a PKC-mediated noncanonical Wnt pathway [40]. However, Wnt7b can mediate high levels of autocrine canonical Wnt/β-catenin activity in pancreatic cancer as well as an anchorage-independent growth phenotype [41]. Therefore, Wnt7b initiates which Wnt pathway depending upon the cell context and further investigations of the underlying role of Wnt7b in bone sarcomagenesis will be required. Wnt11 is a potent inhibitor of β-catenin/TCF activity in many different cell types [42]. Expression of Wnt11 in human carcinoma cells was significantly increased that induces morphological transformation of intestinal epithelial cells [43]. However, Wnt11 levels are low in hepatocellular carcinomas than that in normal liver, leading to a model in which Wnt11 is a tumour suppressor [44]. Our results have illuminated that Wnt11 was remarkably increased in bone sarcoma cells, suggesting Wnt11 as an oncogenic role during bone sarcomagenesis. Selective gain of expression for canonical Wnt ligands and loss of expression for non-canonical Wnt ligands here suggest differential involvement of the canonical and non-canonical Wnt pathways during bone sarcomagenesis. Clearly, further studies are needed to determine the primary function of Wnt6, Wnt7b and Wnt11 in bone sarcomas. Overall, accumulating data presented here and elsewhere in the literature indicate that both canonical and non-canonical Wnt signaling was differentially implicated in bone sarcomagenesis.

Our previous studies have documented that β-catenin was accumulated and increased in chondrosarcoma tissue and SW1353 cells without the exploration of potential mechanisms [23]. In this study, we have presented the evidence of constitutive activation of the canonical Wnt signaling in bone sarcomas involving au autocrine Wnt signaling loop, as evidenced by Wnt antagonists and siLRP6 to downregulate the signaling pathway (Figure 2). Our findings that DKK1 and FRP1, two specific antagonists of Wnt signaling at the level of ligand/receptor interactions [11-14], caused downregulation of active β-catenin levels and TCF transcriptional activity in bone sarcoma cells, strongly implicated an autocrine Wnt signaling loop. Independent evidence in support of this conclusion derived from the use of siRNAs directed against LRP6 that was markedly higher in bone sarcoma cells as shown in Figure 1H. These studies also established that LRP6 was specifically responsible for transducing the Wnt autocrine signal in bone sarcoma cells. Moreover, functional studies have revealed that Wnt antagonists inhibited known Wnt-induced biological effects as well as Wnt target gene expression (Figure 2). In contrast to human colon carcinomas and a variety of other tumors, in which Wnt activation involves genetic mutations in downstream signaling components, APC or β-catenin, resulting in upregulation of this pathway [17, 18], our findings have implied that an autocrine Wnt signaling loop plays a role in the etiology of human bone sarcomas. All these findings are consistent with a model in which bone sarcomas commonly initiate from upregulation of autocrine Wnt signaling, and less frequently from mutations in intracellular components, which established the molecular basis for nuclear accumulation of β-catenin in such bone sarcomas. In addition, the recent report has identified epigenetic silencing of WIF1 in some human osteosarcomas associated with upregulation of Wnt signaling [32]. Thus, whether autocrine Wnt signaling in bone sarcoma cells attributed to Wnt misexpression, aberrant upregulation of other components, or deregulation of Wnt antagonists remains to be elucidated. However, our findings have identified autocrine Wnt signaling loop in bone sarcomas, providing a novel target for therapeutic intervention with DKK1 and FRP1 antagonists or other modalities and aiming interfering with cell surface interactions involving Wnts and their receptors.

Our current study indicates that aberrant Wnt signaling drives the proliferation of bone sarcoma cells and could represent an important step in the pathogenesis of bone sarcomas. As shown in Figures. 3, 4 and 5, stimulation or inhibition by either Wnt3a, GSK-3β inhibitor, or dnTCF4, siLEF4, which affect the canonical Wnt pathway at the distinct levels, had profound effects on the proliferation of bone sarcoma cells. Indeed, stimulation of bone sarcoma cells with Wnt3a led to further nuclear accumulation of active β-catenin, increased levels of β-catenin/TCF transcriptional activity, and activated the downstream target genes, which promoted the proliferation and survival of bone sarcoma cells (Figure 3). All these findings have implied that these bone sarcomas have an intact Wnt signaling pathway and suggest a functional role of the Wnt pathway in the biology of bone sarcomas. Our observations also provide strong support that the Wnt pathway is activated by GSK3β inhibitor, as evidenced by upregulation of active β-catenin and Wnt-responsive reporter activity (Figure 4). These findings were also supported by the previous study that inhibitions of GSK-3β activate β-catenin and attenuate the regulation of the mutant FLT3 receptor on the β-catenin activity in leukemogenesis [45]. Although we cannot exclude that the effect of GSK3β inhibitor acts through other GSK3β-mediated pathways, GSK3β inhibitor induced a robust increase in active form of β-catenin and Wnt-luciferase activity, suggesting that the effect of GSK3β inhibitor occurs, in most part, through activation of the Wnt pathway. Moreover, we also found that GSK3β inhibitor enhanced cell proliferation by attenuating apoptosis. Our findings support the evidence that GSK-3β is recognized as a tumor suppressor, frequently inactivated in a variety of tumors [46]. However, several reports have demonstrated that GSK-3β may actually promote the development of several cancer types, such as oral cancer [46] and osteosarcoma [47]. Therefore, the biological function of GSK-3β in bone sarcomas remained to be elucidated and requires individual assessment in each type of tumors.

Accumulated data suggest that the Wnt signaling exerts its oncogenic functions via upregulation of target genes involved in cell proliferation, exemplified by the β-catenin/TCF transcriptional effects on c-Myc expression in carcinomas [20, 21], and CDC25A recently identified in sarcomas [22]. Actually, we observed that c-Myc is remarkably increased in bone sarcoma cells as compared to hMSC, responsible for Wnt-induced proliferation as previously described [48]. Stimulation of the Wnt/β-catenin by Wnt3a markedly triggered c-Myc expression in bone sarcoma cells, whereas downregulation of β-catenin/TCF signaling resulted in reduction of c-Myc and cyclin D1 levels and decreased cell proliferation. Emerging data suggest that c-Myc expression is frequently overexpressed in bone sarcomas [49], but whether c-Myc plays a central role in Wnt-induced cell proliferation in bone sarcomas remains to be determined. Our findings are partly consistent with the observed effects of TCF downregulation on c-Myc levels in CRC lines mutant for APC or β-catenin [48]. Thus, the high prevalence of Wnt pathway activation in bone sarcoma cells may help to account for the high frequency of c-Myc overexpression in such bone sarcomas. Recently, the authors have identified CDC25A as a Wnt transcriptional target, an important mediator of Wnt-induced sarcoma cell proliferation both *in vitro* and *in vivo* [22]. Therefore, the inhibition of proliferation induced by dnTCF4 or siLEF1 in bone sarcoma cells in the present study might be mediated through c-Myc as well as CDC25A.

In summary, the data presented in this study implicate the aberrant activation of canonical Wnt/β-catenin signaling pathway involving an autocrine Wnt signaling loop in the pathogenesis of bone sarcomas. Autocrine activation of the canonical Wnt/β-catenin signaling drives the proliferation of bone sarcoma cells, whereas antagonism of this pathway decreased the proliferation and induced cell cycle arrest. Our findings have implied that aberrant activation of the Wnt/β-catenin pathway drives the proliferation of bone sarcomas and identify the autocrine Wnt/β-catenin signaling as a potential novel therapeutic target in bone sarcomas.

## MATERIALS AND METHODS

### Cell culture, chemical reagents and antibody

Human osteosarcoma U2OS and Saos-2 cell lines, and chondrosarcoma SW1353 cell line were obtained from the American Type Culture Collection (Bethesda, MD, USA) and grown according to ATCC recommended media and conditions. Human mesenchymal stem cells (hMSCs) were obtained from Lonza and expanded in basal medium according to the manufacturer's instructions. All cells were maintained in a humidified cell incubator with 5% CO_2_ at 37°C.

Recombinant human Wnt3a protein and DKK1 protein were purchased from R&D Systems (Minneapolis, MN). The GSK-3β inhibitor SB-216763 was obtained from Sigma. Antibodies for detection of the following targets were purchased as indicated: total β-catenin and GSK-3β from BD Biosciences, active β-catenin (anti-ABC, clone 8E7) dephosphorylated on Ser37 or Thr41 from Millipore Corporation (Billerica, MA, USA), Cyclin D1 and c-Myc from Santa Cruz Technology, Axin2, TCF4 and LEF1 from Cell Signaling Technology, LRP6 and hnRNP from Abcam, and β-actin from Sigma.

### RNA isolation and Real-time PCR

Total RNA was isolated using Trizol reagent (Invitrogen, Carlsbad, USA). Total RNA (2 μg) was used for the synthesis of first-strand cDNA using M-MLV reverse transcriptase (Invitrogen). Quantitative real-time PCR was performed using the SYBR green mix (Applied Biosystems). The reactions were performed with a 7500 Fast Real-Time PCR System (Applied Biosystems). The data were displayed as 2−ΔΔCt values and were representative of at least three independent experiments. Sequences of the qRT-PCR primers were presented in Table 1.

**Table 1:** Real-time PCR primers for human Wnt/β-catenin signaling family members and downstream genes

Genes	Primer Forward Sequence (5′→3′)	Primer Reverse Sequence (5′→3′)
Wnt1	CGAACCTGCTTACAGACTCCAA	CAGACGCCGCTGTTTGC
Wnt2	GGATGACCAAGTGTGGGTGTAAG	GTGCACATCCAGAGCTTCCA
Wnt3	CTGGGCCAGCAGTACACATCT	GGCATGATCTCGATGTAATTGC
Wnt3a	CCCGTGCTGGACAAAGCT	TCTGCACATGAGCGTGTCACT
Wnt5a	TCTCCTTCGCCCAGGTTGTA	CTTCTGACATCTGAACAGGGTTATTC
Wnt5b	CCAACTCCTGGTGGTCATTAGC	TGGGCACCGATGATAAACATC
Wnt6	TCCGCCGCTGGAATTG	AGGCCGTCTCCCGAATGT
Wnt7b	TGAAGCTCGGAGCACTGTCA	GGCCAGGAATCTTGTTGCA
Wnt8b	AATCGGGAGACAGCATTTGTG	ATCTCCAAGGCTGCAGTTTCTAGT
Wnt10b	CCTCGCGGGTCTCCTGTT	AGGCCCAGAATCTCATTGCTTA
Wnt11	CGTGTGCTATGGCATCAAGTG	GCAGTGTTGCGTCTGGTTCA
Wnt16	GCCAATTTGCCGCTGAAC	CGGCAGCAGGTACGGTTT
Fzd1	CACCTTGTGAGCCGACCAA	CAGCACTGACCAAATGCCAAT
Fzd2	TTTCTGGGCGAGCGTGAT	AAACGCGTCTCCTCCTGTGA
Fzd3	TGGCTATGGTGGATGATCAAAG	TGGAGGCTGCCGTGGTA
Fzd4	GGCGGCATGTGTCTTTCAGT	GAATTTGCTGCAGTTCAGACTCTCT
Fzd5	CGCGAGCACAACCACATC	AGAAGTAGACCAGGAGGAAGACGAT
Fzd6	ACAAGCTGAAGGTCATTTCCAAA	GCTACTGCAGAAGTGCCATGAT
Fzd7	CAACGGCCTGATGTACTTTAAGG	CATGTCCACCAGGTAGGTGAGA
Fzd8	GCTCGGTCATCAAGCAACAG	ACGGTGTAGAGCACGGTGAAC
Fzd9	GCGCTCAAGACCATCGTCAT	ATCCGTGCTGGCCACGTA
Fzd10	GCCGCCATCAGCTCCAT	TCATGTTGTAGCCGATGTCCTT
LRP5	CGTGATTGCCGACGATCTC	TCCGGCCGCTAGTCTTGTC
LRP6	TTATGTGCCACACCCAAGTTCT	CTGAGGGAGCTGATCATTGATTTA
Ror2	GTGCGGTGGCTAAAGAATGAT	ATTCGCAGTCGTGAACCATATT
Ryk	CCCAGGTCAACATTTCTGTTCA	TGCCAGTACAGGAAAGCTCTAC
LRP8	CCCATCCCTAATCTTCACCAAC	CTAGTGCCACGACATTCTTGAG
TCF-1	GGTCCTACGTTCACCAACACA	CTCTGGGTCACATGGCTCT
TCF-3	ACGAGCGTATGGGCTACCA	GTTATTGCTTGAGTGATCCGGG
TCF-4	GGCTATGCAGGAATGTTGGG	GTTCATGTGGATGCAGGCTAC
LEF-1	AGAACACCCCGATGACGGA	GGCATCATTATGTACCCGGAAT
Axin2	CAACACCAGGCGGAACGAA	GCCCAATAAGGAGTGTAAGGACT
c-Myc	GGCTCCTGGCAAAAGGTCA	CTGCGTAGTTGTGCTGATGT
Cyclin D1	GCTGCGAAGTGGAAACCATC	CCTCCTTCTGCACACATTTGAA

### Protein extraction and western blot analysis

Detailed experimental procedures for protein extraction and western blot analysis have been previously described [26]. Briefly, total protein extracts were separated using 12.5% SDS–PAGE, transblotted onto nitrocellulose membranes, and blocked before incubating the membranes with primary antibodies overnight at 4°C. After washing, bound primary antibodies were detected using an HRP-conjugated anti-rabbit IgG or anti-mouse IgG (Sigma). Antibody complexes were detected using Immobilon Western Chemi-luminescent HRP Substrate (Millopore) and exposure to X-Omat film (Kodak).

### Luciferase reporter assays

After treatment, cells plated at 3×10^5^ per well in 6-well plates were cotransfected with 0.2 μg of either the TOP-flash or FOP-flash plasmids (Upstate Biotechnology) and 0.02 μg of the Renilla control plasmid (pRL-TK) utilizing Lipofectamine 2000 (Invitrogen). Cells were harvested at 48 h after transfection, and were lysed and analyzed using the Dual Luciferase Reporter Assay System (Promega, Madison, USA) according to the manufacturer's protocol. Luciferase reporter activity was calculated by dividing the ratio TOP/RL by the FOP/RL ratio.

### Cell proliferation assay

Cells were seeded in triplicate on 96-well plates and incubated with complete medium for 24 h. Quiescent cultures were treated with daily supplemented medium. After treatment, cell proliferation was determined by a Cell Proliferation ELISA, BrdU kit (Roche Applied Science, USA) as per the manufacturer's instructions. Each point represents a mean value and standard of three experiments with three replicates.

### Cell survival assay

SW1353 and U2OS cells were incubated with 200ng/ml Wnt3a for different time points (24, 48, and 72 h). Cells were then added to the MTS solution according to the manufacturer's protocols for growth assay using the MTS kit (Promega) and the absorption was read at 490 nm. Cell survival was calculated as follows: cell survival = absorbance of test group/absorbance of control group.

### Detection of cell apoptosis

SW1353 and U2OS cells were stained with fluorescein isothiocyanate (FITC) labeled AnnexinV that binds to membrane phosphatidylserine and with propidium iodide (PI) that binds to cellular DNA according to the manufacturer's instructions (BD bioscience, USA). Flow cytometry was used to discriminate between intact and apoptotic cells. Briefly, SW1353 and U2OS cells were seeded in 24-well plates (1×10^5^ cells per well) and cultured overnight. After treatment, cells were trypsinized, washed with cold PBS, and resuspended in 100 μl of binding buffer containing 5 μl of FITC-conjugated AnnexinV and 5 μl of PI according to the manufacturer's instructions. After 20 min at room temperature in the dark, 400 μl of binding buffer was added, and samples were immediately analyzed on a FACS Calibur flow cytometer (Becton Dickinson, USA) and analyzed with Cell Quest Software (BD bioscience, USA). The results shown are representative of at least three separate experiments.

### Cell cycle analysis

The percentage of cells in G0/G1, S and G2/M phases was determined by DNA flow cytometry. Briefly, SW1353 and U2OS cells were seeded in 24-well plates (1×10^5^ cells per well) and cultured overnight. After treatment, cells were trypsined, then fixed with 75% ethanol at −20°C for 3 hours. After fixing, cells washed with PBS, then stained with 0.5 ml PI/RNase staining buffer (BD bioscience, USA) for 30 minutes at room temperature in the dark. Cell cycle distribution was analyzed by FACS Calibur flow cytometer (Becton Dickinson, USA) and analyzed for cell cycle phases with ModFit LT 3.2 Software (Verity Software house, USA). The results shown are representative of at least three separate experiments.

### Plasmid, siRNA and transient transfection

FRP1-Flag, DKK1-Flag and dnTCF4 were obtained from Addgene. siLRP6 was obtain from cell signaling technologies. siLEF1 was obtained from Sigma (Target Sequence ATCCCGAGAACATCAAATAAA). Transient transfection of cells was performed using the Lipofectamine 2000 (Invitrogen) as per the manufacturer's instructions.

### Statistical analysis

All experiments were performed independently at least three times, and the results were expressed as the Mean ± SD. Differences between groups were analyzed using the unpaired two-tailed Student's *t*-test. Statistical analysis was performed using SPSS (version 17.0; SPSS, Inc.) and the difference is considered significant when a *P* value is less than 0.05.

## SUPPLEMENTARY MATERIAL FIGURE


