# Supplementary material for: Aberrant activation of Wnt/β-catenin signaling drives proliferation of bone sarcoma cells
Source: Oncotarget. 2015 May 11;6(19):17570–83. doi: 10.18632/oncotarget.4100 (PMC4627329; doi:10.18632/oncotarget.4100)
Supplement: Supplementary file 1 [file oncotarget-06-17570-s001.pdf]

## Aberrant activation of Wnt/β-catenin signaling drives proliferation of bone sarcoma cells

**Supplementary Material**

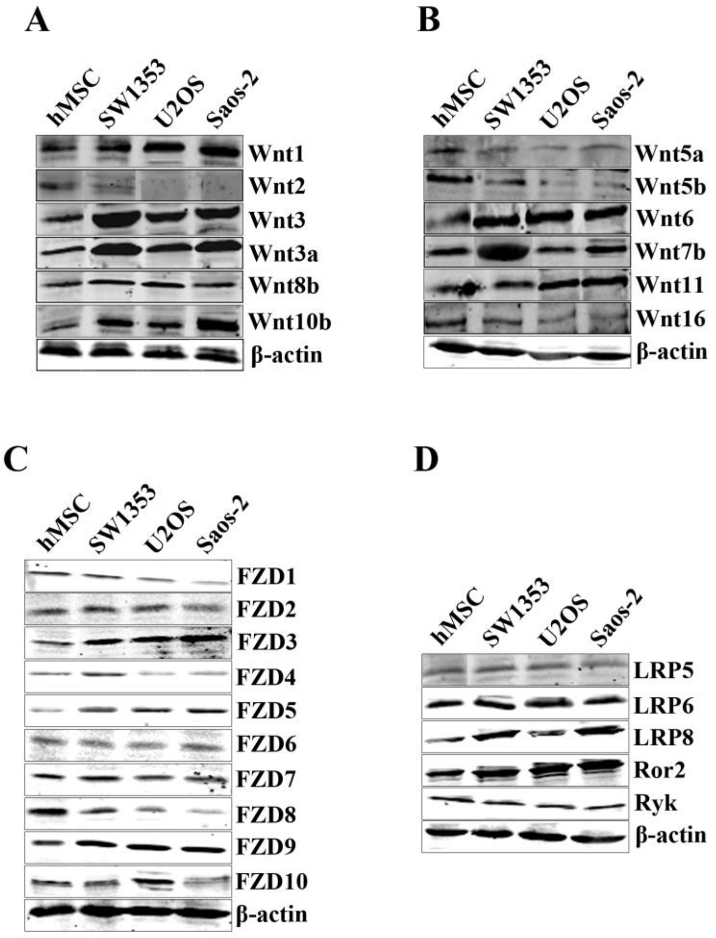


**Figure S1: The protein level of the Wnt signaling components in hMSC and bone sarcoma cell lines**. The relative protein levels of canonical Wnt ligands including Wnt1, Wnt2, Wnt3, Wnt3a, Wnt8b and Wnt10b (A), noncanonical Wnt ligands including Wnt5a, Wnt5b, Wnt6, Wnt7b, Wnt11 and Wnt16 (B), Frizzled receptors such as FZD1-10 (C), Co-receptors such as LRP5, LPR6, LPR8, Ror2 and Ryk (D) were detected by Western blot in hMSC and bone sarcoma cell lines. The whole-cell lysates were immunoblotted with the indicated antibodies. β-actin is used as an internal control.
